# Fermiology and electron dynamics of trilayer nickelate La_4_Ni_3_O_10_

**DOI:** 10.1038/s41467-017-00777-0

**Published:** 2017-09-26

**Authors:** Haoxiang Li, Xiaoqing Zhou, Thomas Nummy, Junjie Zhang, Victor Pardo, Warren E. Pickett, J. F. Mitchell, D. S. Dessau

**Affiliations:** 1 Department of Physics, University of Colorado at Boulder, Boulder, CO 80309 USA; 2 Material Science Division, Argonne National Lab, Argonne, IL 60439 USA; 3 Departamento de Fisica Aplicada and Instituto de Investigacions Tecnoloxicas, Universidade de Santiago de Compostela, Campus Sur s/n, E-15782 Santiago de Compostela, Spain; 4 Department of Physics, University of California, Davis, CA 95616 USA; 5 Center for Experiments on Quantum Materials, University of Colorado at Boulder, Boulder, CO 80309 USA

## Abstract

Layered nickelates have the potential for exotic physics similar to high *T*_C_ superconducting cuprates as they have similar crystal structures and these transition metals are neighbors in the periodic table. Here we present an angle-resolved photoemission spectroscopy (ARPES) study of the trilayer nickelate La_4_Ni_3_O_10_ revealing its electronic structure and correlations, finding strong resemblances to the cuprates as well as a few key differences. We find a large hole Fermi surface that closely resembles the Fermi surface of optimally hole-doped cuprates, including its $d_{x^2-y^2}$ orbital character, hole filling level, and strength of electronic correlations. However, in contrast to cuprates, La_4_Ni_3_O_10_ has no pseudogap in the $d_{x^2-y^2}$ band, while it has an extra band of principally $d_{3z^2-r^2}$ orbital character, which presents a low temperature energy gap. These aspects drive the nickelate physics, with the differences from the cuprate electronic structure potentially shedding light on the origin of superconductivity in the cuprates.

## Abstract (web summary)

Exploration of the electronic structure of nickelates with similar crystal structure to cuprates may shed a light on the origin of high *T*_c_ superconductivity. Here, Li et al. report strong resemblances and key differences of the electronic structure of trilayer nickelate La_4_Ni_3_O_10_ compared to the cuprate superconductors.

## Introduction

Transition metal oxides hold a variety of intriguing electronic phases arising from the strongly correlated *d* electrons. Among these materials, the cuprates have attracted most of the attention, exhibiting compelling physics including the high *T*_C_ superconducting phase, the strange metal scattering rates, pseudogap state, etc^1^. To understand these exotic properties, close analogs of the cuprates have been studied extensively to gain insight into which aspects of cuprate physics are most critical for the superconductivity and other anomalous properties. Layered perovskite nickelates are perhaps the most natural place to look for this physics, as nickel lies directly adjacent to copper in the periodic table, meaning they should also be charge-transfer/Mott insulators^2^ and nickelates can be formed in the same or similar crystal structures as the cuprates^3^. Indeed, some of these materials have been shown to harbor pseudogaps^4^ as well as stripe and checker-board type charge ordering^5, 6^, and recent theoretical works have argued that high temperature superconductivity should likely also present itself^7^. This has motivated recent investigations of LaNiO_3_-based heterostructures and planar-trilayer nickelates that have been designed to imitate the electronic configuration of cuprates in pursuit of potential high *T*_C_ superconductivity and other intriguing cuprate-related phenomena^3, 8–11^.

Unlike the single-layer nickelate compounds, where a metallic state is difficult to obtain, or non-layered structures (such as LaNiO_3_) which are not superconducting even in the cuprates, trilayer Ruddlesden–Popper nickelates R_4_Ni_3_O_10_ (R = La, Pr), with formal oxidation state 2.67 + (*d*^7.33^), are good metallic materials of correlated electrons that are natural for detailed study. However, such studies have not been possible until now because of the difficulty of preparing high-quality single crystal samples. This has recently been overcome for trilayer nickelates through the use of special high-pressure floating-zone image furnaces, which now enable single crystals of these nickelates to be prepared^10^. In the planar-trilayer nickelate La_4_Ni_3_O_8_, recent studies^10, 12^ have revealed stripe charge ordering with a phase transition at 105 K. As for La_4_Ni_3_O_10_, a charge–density wave instability has been predicted below the metal-to-metal transition around 140 K^13, 14^.

In this paper, we present the electronic structure and dynamics of trilayer La_4_Ni_3_O_10_ using angle-resolved photoemission spectroscopy (ARPES) and compare our observations on La_4_Ni_3_O_10_ to cuprates and to density functional theory (DFT) band structure calculations. Our ARPES measurements reveal a gapless hole pocket that resembles the hole Fermi surface of cuprates. Comparing our data to the DFT calculation, most parts of the La_4_Ni_3_O_10_ Fermi surface present a mass enhancement of 2–2.5, which is very similar to what is observed in cuprates in the normal state. On the other hand, an extra hole pocket with strong $d_{3z^2-r^2}$ orbital character exhibits a flat band dispersion near the Fermi level, revealing a 20 meV energy gap that shows a connection to the transition found in the resistivity curve. Our study on La_4_Ni_3_O_10_ will help resolve the anomalous physics of the nickelates (such as the resistivity curve and any potential ordering tendencies associated with it), while also revealing the connection to the cuprate physics. This therefore provides insight into the potential testing ground for cuprate-like properties, as well as articulating the electronic structure of this novel nickel-based oxide.

## Results

### Electronic structure of La_4_Ni_3_O_10_

**Fig. 1:**
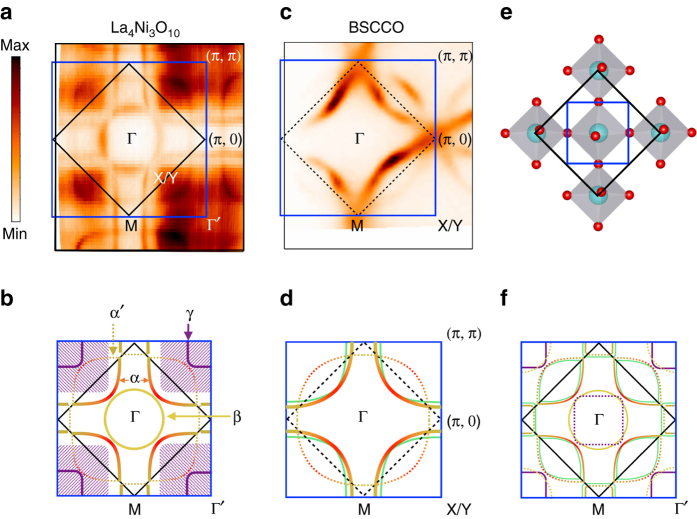
Fermi surface map of La_4_Ni_3_O_10_. **a**, **b** The unsymmetrized Fermi surface of La_4_Ni_3_O_10_ from ARPES measurement and schematic of the Fermi surface. **c**, **d** Fermi surface of optimally doped cuprate (Bi,Pb)_2_Sr_2_CaCu_2_O_8+δ_ (BSCCO) and a schematic of its Fermi surface. The Fermi surface of La_4_Ni_3_O_10_ can be divided into three parts. The hole pocket centered at Γ′ is similar to the hole-doped cuprate Fermi surface (*solid red–yellow curve*) with the (π, π) back-folded band. This cuprate-like hole pocket corresponds to 17% of hole filling level. The other two parts are the extra electron (*yellow circle* in **b**) and hole (*purple curve* and *hatched area*) pocket around Γ and Γ′, respectively. **e** The real space unit cells of La_4_Ni_3_O_10._ The *black box* corresponds to the two Ni unit cells where the *blue box* corresponds to the origin Ni-O plaquette. **f** Drawing of the Fermi surface in the repeated zone representation where pockets are back folded into the small Brillouin zone. The *light green* curves in **d** and **f** represent the extra band expected from multilayer band splitting

The crystal structure of the Ni-O plane in La_4_Ni_3_O_10_ is depicted in Fig. 1e. Due to the out-of-plane tilted Ni-O octahedra, the original Ni-O plaquette (*blue box*) is reconstructed into a two Ni unit cell (*black box*) with double the volume, which is similar to the reconstruction of the antiferromagnetic unit cell in the cuprates. Panels a and b of Fig. 1 show the Fermi surface of La_4_Ni_3_O_10_ taken at 30 K and the corresponding schematic drawing. The original Brillouin zone that corresponds to the Ni-O plaquette is marked as the *blue box*, while the *black box* corresponds to the folded Brillouin zone of La_4_Ni_3_O_10_. Different parts of the Fermi surface are highlighted with different colors and marked as α, α′, β and γ in Fig. 1b. In resemblance to the cuprate ((Bi,Pb)_2_Sr_2_CaCu_2_O_8+δ_) Fermi surface antibonding band shown in Fig. 1c, d, La_4_Ni_3_O_10_ also displays a large hole pocket centered at the corner of the original Brillouin zone (α band). Due to the doubling of the unit cell, this cuprate-like hole pocket (α band) is (π, π) back-folded, with the back-folded bands drawn as *dashed curves* (α′ band) in Fig. 1b. This back-folded feature resembles the shadow band observed in the cuprate Fermi surface^15–18^. In Fig. 1f, we sketch the reconstructed Fermi surface with all the back-folded bands shown as *thin dotted curves*. However, the measurement only reveals the β pocket (*yellow circle*) at the original Brillouin zone center while the γ pocket (*purple*) is only present at the original Brillouin zone corner. This absence of a strong back-folded band feature from the broken translation symmetry of the crystal has been observed in many other materials and investigated in previous studies^19–21^. When the folding potential from the broken translation symmetry is weak, the spectral intensity of the back-folded bands is also expected to be extremely weak, and sometimes further reduced for symmetry reasons^21^—therefore, it is not surprising that these back-folded bands are not observed. Here we will show from both polarization-selective ARPES experiments and DFT calculations that the orbital character of the cuprate-like hole pockets possess a strong $d_{x^2-y^2}$ weighting (similar to cuprates), while the other parts of the Fermi surface (not present in cuprates) display a $d_{3z^2-r^2}$ dominant orbital character. Details of the polarization-dependent ARPES experiment and orbital characters are discussed in Supplementary Note 1 and Supplementary Fig. 1. Counting the size of the cuprate-like hole pocket in Fig. 1a, it occupies ~58.5% of the original Brillouin zone. With the scheme of 1 + p holes per nickel per degenerate spin direction, this indicates the hole doping level corresponding to this part of the Fermi surface is ~17%, similar to the optimally hole-doped cuprates. The γ pocket with a $d_{3z^2-r^2}$ orbital character centered around the zone corner shows a blurry outline with the spectral weight covering a large volume of the Brillouin zone, which is depicted as the hatched area in the schematic drawing (Fig. 1b). This blurry spectral weight exhibits a stark contrast to other parts of the Fermi surface. As we will show later, this blurry spectral weight is due to a flat band lying extremely close to the Fermi energy over a large region of *k*-space.

**Fig. 2:**
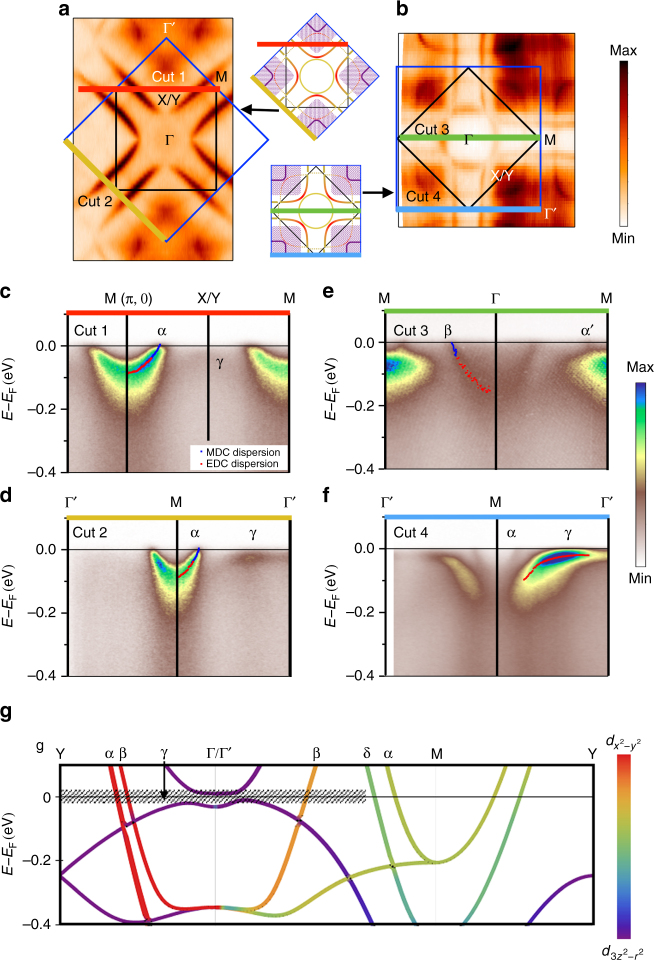
Fermi surface maps and high symmetry cuts of La_4_Ni_3_O_10_ measured at low temperature (*T* = 30 K). **a**, **b** Fermi surface maps of La_4_Ni_3_O_10_ taken with different experimental geometries, where map **a** emphasizes the cuprate-like hole pocket (α band), while map **b** shows the comprehensive structure. The *blue* and *black boxes* are the unfolded and folded Brillouin zones, respectively. **c**–**f** ARPES spectra of the high symmetry cuts. Each high symmetry cut position is indicated as the *colored line* in **a**, **b**. Cut 4 is taken along Γ-M in the second Brillouin zone to avoid the weak matrix element in the first Brillouin zone. The *dots* in **c**–**f** depict the MDCs (*blue*) and EDCs (*red*) peak position, which indicate the band dispersion. **g** Band structure from DFT calculation under the folded Brillouin zone. The weightings of $d_{3z^2-r^2}$ and $d_{x^2-y^2}$ orbital character are shown with different color scales. The *hatched area* indicates the blurry area from the γ band spectral weight in the Fermi surface

In Fig. 2, we present additional aspects of the electronic structure utilizing two different experimental geometries, taking advantage of the photon polarization/matrix element effects to highlight various features of the data, particularly the $d_{x^2-y^2}$ and $d_{3z^2-r^2}$ bands (see Supplementary Note 1 and Supplementary Fig. 1 for more details). In cuts 1 and 2 (Fig. 2c, d), which are the high symmetry cuts through the Fermi surface shown in Fig. 2a, we observe a sharp band dispersion near the M point corresponding to the cuprate-like hole pocket near the boundary of the unfolded Brillouin zone. This region in momentum space resembles the antinodal region in the hole-doped cuprates, where the band structure comes to a saddle point at (π, 0) roughly around −100 meV, and hosts the largest energy gap in both the superconducting and pseudogap phases. Just as in the cuprates, the band shown here for La_4_Ni_3_O_10_ comes to a saddle point at (π, 0) near −100 meV, shown for both cuts 1 and 2 of Fig 1. To quantify the dispersion, we have extracted peak positions from both energy distribution curves (EDCs) and momentum distribution curves (MDCs), with the extracted peak positions plotted as *red* and *blue dots* respectively, in each ARPES spectrum.

Other aspects of the electronic structure can be observed by a 45-degree azimuthal rotation of the sample relative to the incident direction of the photons (Fig. 2b), giving selection rules that highlight different symmetry states from those of Fig 1b. In cut 3, a slice through the electron pocket (β band) displays a clear dispersion near the Fermi level. However, the ratio of coherent spectral weight to the background weight is not so strong for this band. In cut 4, around the Γ′ point, a cut through the γ pocket with $d_{3z^2-r^2}$ orbital character displays a spectral weight that is broadened near the Fermi level. The dispersion of the γ band is flattened near the Fermi level indicating exotic low-energy electron dynamics. This band behaves quite differently from the α and β bands that are highly dispersive across the Fermi level.

We calculated the band structure using the all-electron, full-potential code WIEN2k^22^ based on the augmented plane wave plus local orbital (APW + LO) basis set^23^. As exchange-correlation potential we have used the generalized gradient approximation in the Perdew–Burke–Ernzerhof scheme^24^. The results of these calculations are plotted in Fig. 2g. The α, β, and γ bands are labeled in the plot, which correspond to the three different parts of the Fermi surface observed experimentally. Along the Γ/Γ′-M direction, there is an extra *green band* (δ) that crosses the Fermi level that has not been experimentally resolved in our data. In the calculation, the δ band (*green*) and the α band (*yellow*) originate from the outer and inner Ni-O planes, respectively. In the experimental data (Fig. 2d) only one band in this direction is resolved, whose Fermi momentum (**k**_**F**_) coincides closely with the one from the inner Ni-O planes, i.e., the α band (see details in Supplementary Fig. 2). We discuss more details about the missing band splitting in the experimental data later in the paper.

The color scale that indicates the orbital weighting shows a dominant $d_{x^2-y^2}$ orbital character for the cuprate-like hole pocket (α band), while the γ hole pocket reveals a dominant $d_{3z^2-r^2}$ orbital character. As for the β band, the DFT calculation exhibits a mixture of $d_{x^2-y^2}$ and $d_{3z^2-r^2}$ orbital character. This theoretical result is consistent with our polarization-dependent ARPES result in Supplementary Fig. 1.

### Band renormalization and mass enhancement

**Fig. 3:**
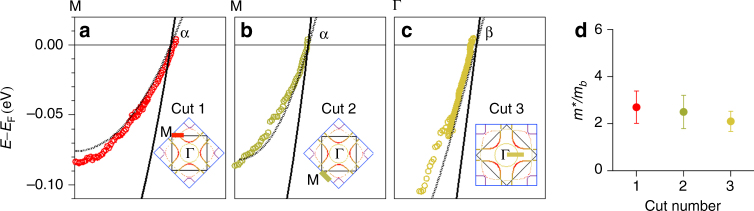
Low-energy electron dynamics. **a**–**c** Experimental band dispersions (*colored dots*), where the cut positions are indicated in the insets. The *solid black curves* are the calculated dispersions from DFT theory for the relevant bands. The *dotted black curves* are the DFT bands scaled by the corresponding mass enhancement values. **d** The measured mass enhancements of each band, which are all roughly 2–2.5. The *error bars* denotes the s.d. of the mass enhancement determined by using various ranges of the band dispersion

The measured band dispersions from EDCs and MDCs analyses of the data of Fig. 2 are shown in Fig. 3a–c (*open colored circles*) and are compared to the results from our DFT calculation (*black lines*). It is seen that the measured dispersions are flatter than the calculated ones, implying a mass-renormalization (self-energy) effect. We extracted the mass enhancements from the ratio of the second derivatives of the measured to calculated dispersions, which further quantifies the band renormalization effect. The values of mass enhancements in *panel* d of Fig. 2 are around 2.2 for each of the bands, which are similar to results of cuprates in the normal state, which are also around 2^25–27^. The *dotted curves* in Fig. 3a–c are the DFT bands scaled by the mass enhancement value, and show good agreement with the measured band dispersions. We do not show the γ band here due to an energy gap opening in this band—details will be discussed later.

### Gapped and non-gapped portions of the Fermi surface

**Fig. 4:**
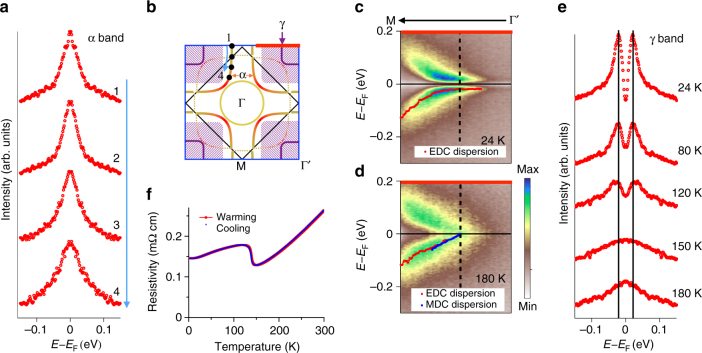
Temperature evolution of energy gaps. **a** Symmetrized EDCs at multiple **k**_**F**_ positions of the α band at 30 K. **b** Schematic of the Fermi surface. The *black dots* indicate the **k**_**F**_ positions of the symmetrized EDCs in **a**. **c**, **d** Spectra at 24 and 180 K. **k**_**F**_ is indicated by the *dashed line*. The *thick red line* in **b** shows the position of the cuts in both **c** and **d**. The *dots* in the spectra are the EDC and MDC peak positions that sketch out the band dispersion. The band dispersions near *E*_F_ flatten at 24 K but linearly disperse up to *E*_F_ at 180 K. **e** Symmetrized EDCs at multiple temperatures. **f** Resistivity curve of La_4_Ni_3_O_10_. The resistivity anomaly at ~140 K is consistent with the energy gap filling in between 120 and 150 K

To further understand the low-energy electron dynamics, we investigate the energy gap of two different bands. For the cuprate-like hole pocket, symmetrized EDCs at multiple **k**_**F**_ points measured at 30 K are plotted in Fig. 4a, with *k*-locations labeled in Fig 4b. Using the standard method typically applied to cuprates^28^, the single peaks at the Fermi level for each of the points indicate a state that is ungapped. Unlike the single-layer nickelate, where a pseudogap is observed near the antinodal region^4^, the cuprate-like band in this trilayer nickelate shows no energy gap opening. However, for the γ band with $d_{3z^2-r^2}$ orbital character, symmetrized spectrum taken at 24 K (Fig. 4c) shows a dip of spectral weight at the Fermi level and the band dispersion turns flat and extends to a wide range in momentum when approaching the Fermi level. These features reveal a significant energy gap of about 20 meV in this band. This is consistent with our observation in the low temperature Fermi surface, in which the hole pocket covers a blurry area where no well-defined Fermi momentum can be observed. However, in the 180 K spectrum (Fig. 4d), the coherent spectral weight displays a linear band dispersion across the Fermi level. The symmetrized EDCs in Fig. 4e describe more temperature-dependent behavior of this electronic dynamics. When moving toward higher temperature, the spectral weight within the energy gap gradually increases and the coherence peaks of the spectra are broadened. The disappearance of the energy gap between 120 and 150 K is consistent with the resistivity curve in Fig. 4f, which display the anomaly at ~140 K. This indicates the likely connection of the energy gap opening to the phase transition found in the resistivity curve.

## Discussion

In general, the electronic structure and dynamics of La_4_Ni_3_O_10_ present multiple commonalities to the high *T*_C_ superconductor cuprates. First, the Fermi surface of La_4_Ni_3_O_10_ reveals a hole pocket with $d_{x^2-y^2}$ orbital character that is in many ways similar to the hole surface in cuprates. The volume of this hole part of the Fermi surface indicates a doping level of 17%, close to the optimal hole doping for superconductivity in cuprates. Second, the mass enhancement of La_4_Ni_3_O_10_ shows a similar value to that of the normal state of the cuprates^25–27^. The similarity of this renormalization effect in La_4_Ni_3_O_10_ and cuprates indicates a similarity of the electronic correlations in these two materials. Third, the back-folded band of the cuprate-like hole pocket in La_4_Ni_3_O_10_ resembles the shadow band feature observed in the Fermi surface of cuprates. The origin of this shadow band phenomenon in cuprate is controversial^15–18^. It is believed to originate from the antiferromagnetic correlation or a structural distortion of the crystal. However, in La_4_Ni_3_O_10_, this feature most likely arises from the broken translation symmetry due to the tilted Ni-O octahedra.

In addition to these commonalities with the cuprates mentioned above, there are some unique properties observed in this material.

First, the cuprate-like hole pocket in La_4_Ni_3_O_10_ reveals no energy gap opening, unlike the underdoped multilayer cuprates^29^ or the single-layer nickelate (Eu_0.9_Sr_1.1_NiO_4_) that both host pseudogap states^4^. The general trend of a pseudogap in the single-layer compound and smaller or absent pseudogap in higher dimensionality compounds (bilayer, trilayer, and infinite-layer perovskites, respectively) is fully consistent with the trends observed in other doped Mott insulators, including the cuprates^29, 30^, iridates^31, 32^, and ruthenates^33^, even though the origin of the pseudogap in all of these compounds remains controversial.

Second, the band splitting expected from the multilayer coupling of this trilayer material is unresolved in our ARPES data (Fig. 2; Supplementary Fig. 2). This is in contrast to trilayer cuprate (Bi_2_Sr_2_Ca_2_Cu_3_O_10+δ_) that has revealed band splitting^34^. Some other layered transition metal oxides have also revealed clear band splitting from bilayer coupling^35, 36^. We consider two explanations why our experimental data does not show this band splitting. (A) The band splitting may exist as calculated, but one of the two split bands is greatly reduced in intensity, possibly due to the matrix element effect, which is a combination of different photon energies, polarizations, and experimental geometries (see Supplementary Fig. 3 and Supplementary Note 3 for a detailed photon energy scan). In this scenario, we argue that the α band observed in our ARPES data is due to the inner-plane nickel band from DFT (Fig. 3a, b; Supplementary Fig. 2), since the **k**_**F**_ position of these two bands is extremely similar, leaving the outer nickel band (δ band in Fig. 2g) unresolved. For Luttinger counting of the Fermi surface, this unresolved δ band should exist in order to give a correct electron filling in the system. (B) The actual band splitting might be much weaker than expected from the DFT result, and if the splitting is comparable with the energy or momentum widths of the constituent states, the splitting will become unresolvable. Such a situation has previously been discussed in both bilayer^37^ and trilayer^38^ cuprates. In this case, we would nominally expect that the centroid of the unresolved states would be at the calculated centroid of the split bands rather than at one of them, which is inconsistent with our experimental observation. On the other hand, the centroid of the non-split bands could be at a slightly different *k*-value if other states took up the required number of electrons missing from these bands, with the γ band being the most natural candidate; because it skims right along the Fermi surface, it can accommodate a large number of electrons with a minimal change in chemical potential. Further investigation is required to resolve this delicate band splitting issue.

Third, the extra $d_{3z^2-r^2}$ character band in this material raises intriguing issues. The flat band dispersion of the γ hole pocket at low temperature shows a strong spectral weight right below the Fermi level (Fig. 4) and its residual spectral weight at the Fermi level covers a large area in *k*-space. The energy gap observed in this band exhibits a sharp coherence peak. For the pseudogaps in cuprates and some other transition metal oxides, the depletion of spectral weight instead of a coherent gap characterizes the opening of the pseudogap^4, 31, 39, 40^. In this regard, the origin of this energy gap in La_4_Ni_3_O_10_ may differ from the commonly known pseudogap state. On the other hand, the coincidence of the gap evolution with the resistivity anomaly implies the connection to the potential charge–density waves in this material^13, 14^. However, the spread of the γ band in *k*-space especially at low energy near the Fermi level implies it to be agnostic to certain nesting q vectors and the corresponding charge modulations. To fully determine the charge–density wave origin, further studies of the electronic structure combined with other experimental techniques like scanning tunneling spectroscopy and X-ray diffraction are required and are beyond the scope of this paper.

Fourth, the band top of the γ hole pocket is relatively close to the Fermi level and the band dispersion is extremely flat near *E*_F_, which makes it susceptible to undergoing a Lifshitz transition that can lead to topology changes as well as a drastic change of carrier density on the Fermi surface. The Lifshitz transition has been a topic of intense discussion in various materials^41–43^ and has been recently argued to have a close connection to superconductivity in layered iron-based superconductors^43–48^. The susceptibility to a Lifshitz transition in the γ hole pocket with minor changes of the chemical potential or band curvature gives the potential for fine tuning the electronic structure and topology in these compounds.

In summary, we present a comprehensive study of the electronic structure and dynamics of the trilayer nickelate (La_4_Ni_3_O_10_). Our work reveals a hole pocket that resembles the cuprate hole Fermi surface and displays similar renormalization effects. These similarities in both electronic structure and dynamics imply a possibility that more cuprate properties may be achievable in this material including high *T*_C_ superconductivity. On the other hand, we found an extra $d_{3z^2-r^2}$ orbital band that displays an energy gap opening coinciding with the phase transition observed in the transport measurement. To separate the $d_{3z^2-r^2}$ orbital band from the cuprate-like hole pocket, future developments such as changing the carrier density by doping, or changing the uniaxial pressure with different layer-spacing elements may help to move the $d_{3z^2-r^2}$ orbital band away from the Fermi level, bringing the electronic structure of these materials even closer to that of the cuprates.

## Methods

### Crystal growth and transport measurement

Single crystal growth of La_4_Ni_3_O_10_ was performed in an optical-image floating zone furnace (HKZ-1, SciDre GmbH) with 20 bar O_2_. Resistivity of La_4_Ni_3_O_10_ single crystals (Fig. 4f) was measured on a Quantum Design PPMS in the temperature range of 1.8–300 K using a conventional four-probe method with contacts made with silver paint.

### ARPES measurements

ARPES experiments were carried out at the Advanced Light Source using beamlines 4.0.3, 10.0.1, and 7.0.2. The energy resolution was 13 meV at beamline 7.0.2 and 20 meV at beamline 4.0.3. All data shown in the paper were measured with the photon energy of 75 eV unless otherwise noted. The Fermi surface maps in Fig. 2a and the ARPES spectrum in Fig. 2c, d were taken at beamline 4.0.3. The ARPES data shown in Fig. 1a, Fig. 2b, e, f, and Fig. 4 were taken at beamline 7.0.2. All Fermi surface maps shown in the paper are integrated intensity over *E*_F_ ± 5 meV. The polarization-dependent study was carried out at beamline 10.0.1 with glancing incident photons. Results of this are shown in Supplementary Note 1 and Supplementary Fig. 1.

### Data availability

The data that support the plots within this paper and other findings of this study are available from the corresponding author on reasonable request.

## Electronic supplementary material

Supplementary Information
