# Author Correction: Fermiology and electron dynamics of trilayer nickelate La_4_Ni_3_O_10_

**DOI:** 10.1038/s41467-018-04105-y

**Published:** 2018-05-11

**Authors:** Haoxiang Li, Xiaoqing Zhou, Thomas Nummy, Junjie Zhang, Victor Pardo, Warren E. Pickett, J. F. Mitchell, D. S. Dessau

**Affiliations:** 1 Department of Physics, University of Colorado at Boulder, Boulder, CO 80309 USA; 2 Material Science Division, Argonne National Lab, Argonne, IL 60439 USA; 3 Departamento de Fisica Aplicada and Instituto de Investigacions Tecnoloxicas, Universidade de Santiago de Compostela, Campus Sur s/n, E-15782 Santiago de Compostela, Spain; 4 Department of Physics, University of California, Davis, CA 95616 USA; 5 Center for Experiments on Quantum Materials, University of Colorado at Boulder, Boulder, CO 80309 USA

Correction to: *Nature Communications* 10.1038/s41467-017-00777-0, published online 26 September 2017

The original version of this Article contained errors in Fig. 2g, Fig. 3a–c and Supplementary Fig. 2. In Fig. 2g and Supplementary Fig. 2, the band structure plot calculated from density function theory (DFT) had a missing band of mainly *z*^2^ character that starts at about – 0.25 eV at the *Y* point and disperses downwards towards the Γ point. This band was inadvertently neglected when transferring the lines from the original band plot to the enhanced version for publication. Also in Fig. 2g, the points labelled *M* and *Y* were not exactly at (1/2 1/2 0) and (0 1/2 0), but rather (0.52 0.48 0) and (0 0.48 0) due to a bug in XCrysDen for low-symmetry structures that the authors failed to identify before publication. Thus, the bands presented were slightly off the true *M*–*Y* direction and additional splitting incorrectly appeared (in particular for the highly dispersive bands of *x*^2^–*y*^2^ character). The correct versions of Fig. 2g (cited as Fig. 1) and Supplementary Fig. 2 (cited as Fig. 2) are:

**Fig. 1:**
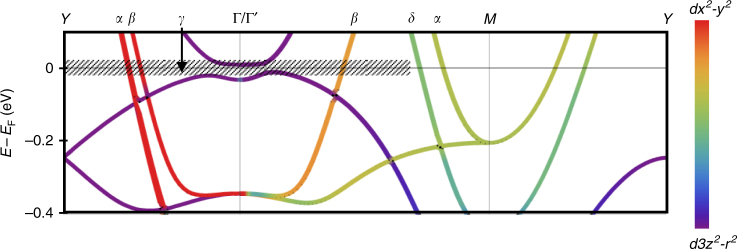
.

**Fig. 2:**
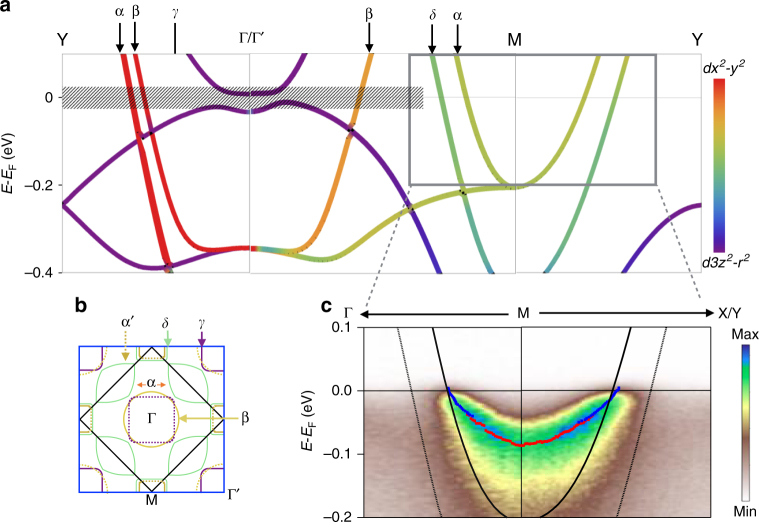
.

which replaces the previous incorrect version, cited here as Fig. 3 and Fig. 4:

Neither of these errors in Fig. 2g or Supplementary Fig. 2 affects either the discussion or any of the interpretations of the ARPES data provided in the paper. The authors discussed the multilayer band splitting along the Γ–*M* direction (*δ* band and *α* band as assigned in the paper), and ARPES did not see any split band. The authors did not discuss the further splitting that arises due to back folding along the *M*–*Y* direction.

**Fig. 3:**
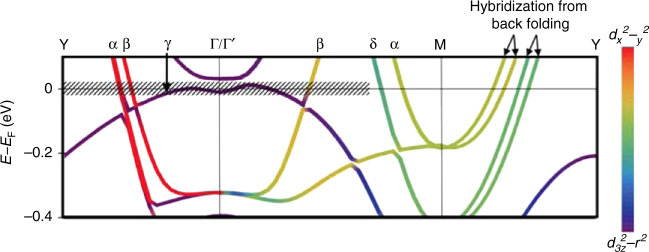
.

**Fig. 4:**
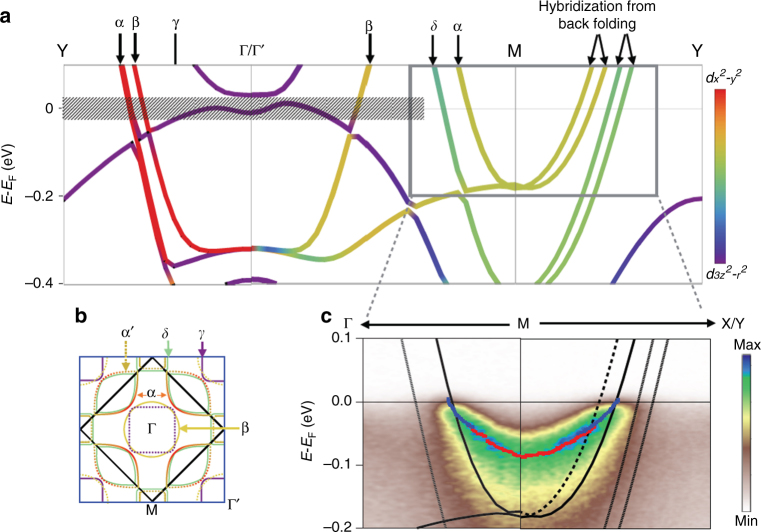
.

In Fig. 3a–c, the errors in the *M* and *Y* points in Fig. 2g cause subtle changes to the DFT dispersions. The correct version of Fig. 3a–c is cited here as Fig 5:

**Fig. 5:**
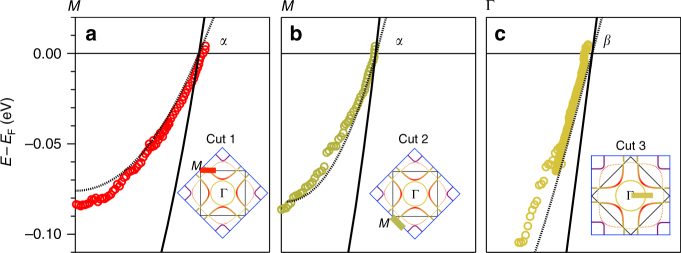
.

**Fig. 6:**
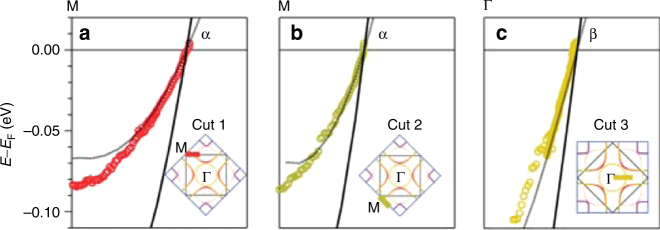
.

which replaces the previous incorrect version (Fig. 6):

However, the influence on the effective mass results of Fig. 3d is negligible.

These errors have now been corrected in both the PDF and HTML versions of the Article. The authors acknowledge James Rondinelli and Danilo Puggioni from Northwestern University for calling our attention to these issues.

