# Addendum: Fermiology and electron dynamics of trilayer nickelate La_4_Ni_3_O_10_

**DOI:** 10.1038/s41467-018-04106-x

**Published:** 2018-05-16

**Authors:** Haoxiang Li, Xiaoqing Zhou, Thomas Nummy, Junjie Zhang, Victor Pardo, Warren E. Pickett, J. F. Mitchell, D. S. Dessau

**Affiliations:** 1 Department of Physics, University of Colorado at Boulder, Boulder, CO 80309 USA; 2 Material Science Division, Argonne National Lab, Argonne, IL 60439 USA; 3 Departamento de Fisica Aplicada and Instituto de Investigacions Tecnoloxicas, Universidade de Santiago de Compostela, Campus Sur s/n, E-15782 Santiago de Compostela, Spain; 4 Department of Physics, University of California, Davis, CA 95616 USA; 5 Center for Experiments on Quantum Materials, University of Colorado at Boulder, Boulder, CO 80309 USA

**Keywords:** Electronic properties and materials, Superconducting properties and materials

Addendum to: *Nature Communications* 10.1038/s41467-017-00777-0; published online 26 September 2017

**Supplementary Note 4 | Structural information of the DFT calculation**

The DFT calculations in the main text use crystal structure information determined by x-ray diffraction (XRD) measurements of La_4_Ni_3_O_10_. The room temperature XRD measurements on all samples were consistent with mixed phases of orthorhombic and monoclinic symmetry. However, the in-plane lattice constants of these two phases differ by <1%, leading to minimal influence on band structure calculations. Furthermore, any impact on the experimental Fermi surface attributable to such deviations would be obscured by broadening of the bands measured by ARPES. Therefore, the DFT calculations adopt the higher symmetry orthorhombic crystal structure with space group *Cmca* and lattice constants *a* = 5.417 Å, *c* = 5.468 Å, *b* = 27.962 Å, with some additional relaxation of the internal coordinates. The detailed atomic coordinates are presented in Supplementary Table 1.

**Table 1:** Supplementary Table 1 | The detailed atomic coordinates from the DFT calculation

Atom	Wyckoff position	Coordinates
**La1**	8f	(0, 0.4326, 0.0014)
**La2**	8f	(0, 0.3016, 0.0099)
**Ni1**	4a	(0, 0, 0)
**Ni2**	8f	(0, 0.1394, 0.0024)
**O1**	8e	(0.25, 0.4921, 0.25)
**O2**	8f	(0, 0.0704, 0.0501)
**O3**	8e	(0.25, 0.3664, 0.25)
**O4**	8f	(0, 0.2161, 0.9641)
**O5**	8e	(0.25, 0.1461, 0.25)

A comprehensive plot of the DFT results for this orthorhombic structure is shown in Supplementary Figure 4.

**Figure:**
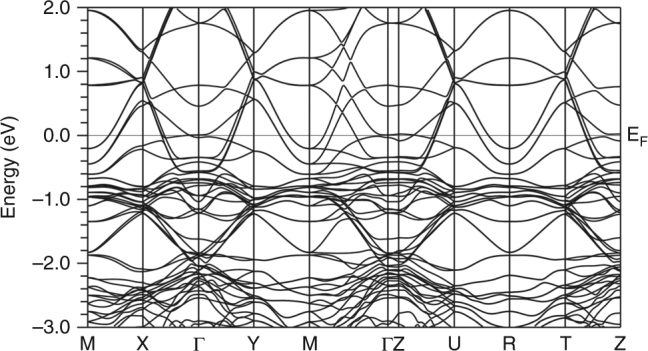
Supplementary Figure 4 | DFT band structure of orthorhombic La_4_Ni_3_O_10_ along more directions and over a wider energy range than is shown in the main text

