# Supplementary material for: Fermiology and electron dynamics of trilayer nickelate La4Ni3O10
Source: Nat Commun. 2017 Sep 26;8:704. doi: 10.1038/s41467-017-00777-0 (PMC5614968; doi:10.1038/s41467-017-00777-0)
Supplement: Supplementary file 1 — Supplementary Information [file 41467_2017_777_MOESM1_ESM.pdf]

**Supplementary Note 1**

**Polarization-dependent ARPES experiment and orbital characters**

Polarization-dependent ARPES has been proved to be a powerful experimental tool to identify the orbital symmetry in electronic structure [1,2,3,4]. To understand the orbital characters in different parts of the Fermi surface in $La_4Ni_3O_{10}$, we performed polarization-dependent ARPES measurement on this material. A schematic drawing of the experimental geometry is sketched in Supplementary Fig. 1a. In the first row of the figure (panels b, c) taken along the green cut of the schematic, one can observe a stark contrast between the s and p polarization. In the s polarization, the nodal-cut of the cuprate-like hole pocket shows a band with good contrast, while the same band disappears in the p polarization. The other hole pocket shows an opposite behavior, where it disappears in the s polarization but exhibits high intensity in the p polarization. The electron pocket around Γ (yellow circle in Supplementary Fig. 1a) is difficult to resolve in Supplementary Fig. 1b,c as the spectral intensity is contaminated by the other two bands. In order to resolve the band dispersion of this electron pocket, we take a slightly different cut along the blue line in Supplementary Fig. 1a, with the band dispersion sketched as the yellow curve in the spectra (β band, Supplementary Fig. 1d, e), The contrast of this band with the two polarizations is not as sharp as the other band in Supplementary Fig. 1, as it shows a roughly equal intensity in both polarizations.

The table in panel f describes the parity of the polarization vectors and the orbitals with respect to the mirror plane. The matrix element for the in-plane $d_{x^2-y^2}$ orbital is strong in the s polarization but weak in the p polarization while the $d_{3z^2-r^2}$ orbital has an opposite trend. Thus, we can conclude that the cuprate-like hole pocket (α band, orange) exhibits a

$d_{x^2\text{-}y^2}$ orbital character whereas the γ hole pocket around Γ′ (γ band, purple) reveals a $d_{3z^2\text{-}r^2}$ orbital character and the β electron pocket around Γ point (β-band, yellow) shows a mixture of these two orbitals.

## Supplementary Note 2

### Band splitting around M point

Around the M point in the Supplementary Fig. 2 a, there are more bands that cross the Fermi level than what we observe in our measurement. In Supplementary Fig. 2c, the ARPES spectrum is overlaid with the theoretical band dispersion around the M point. The calculated outer nickel bands do not coincide with any band dispersion in the spectrum at $\mathbf{k_F}$, whereas the inner nickel bands show a good match. In Fig. 3 in the main text, we chose the bare bands from the DFT calculation that have the best fit of Fermi momentum with band dispersion in the measurement.

## Supplementary Note 3

### Photon energy scan

To further investigate the possibility of multilayer splitting, we explored the matrix element effect with different photon energies and polarizations near the M point (Supplementary Fig. 3). Supplementary Fig. 3b shows the stack of Momentum Distribution Curves (MDCs) at $E_F$ at many different photon energies – every eV from 50 eV to 90 eV. In this set of data, the M point and the $\mathbf{k_F}$ position of the α band are represented by the blue solid line and the dashed yellow line respectively, whereas the green dotted line shows the expected $\mathbf{k_F}$ position of the δ band from the outer Ni-O

planes, as predicted by our DFT calculation. Though there are quite strong modulations of the ARPES intensity with photon energy, the two main peaks of the experimental MDCs match the expected $\mathbf{k_F}$ positions for the $\alpha$ band for every energy where a peak is resolvable. Further, there is never a clear peak along the green dotted lines where we expect the $\delta$ band to reside. This is consistent with all of the other data in the paper, though we also note that it is difficult to completely exhaust the potential matrix element effect in the experiment because of the huge potential parameter space.

We also consider the possibility that the outer Ni-O plane, which is expected to give rise to the $\delta$ band, might be affected by the surface potential in such a way as to move its k-position towards the $\alpha$-band or to greatly reduce its intensity. That the $\delta$-band should be more sensitive to the surface than the other bands is plausible since the outer Ni-O plane is closer to the cleaved surface, which is expected to be between the La-O planes (Supplementary Fig. 3c).

**Supplementary Note 4**

**Structural information of the DFT calculation**

The DFT calculations in the main text use crystal structure information determined by x-ray diffraction (XRD) measurements of $La_4Ni_3O_{10}$. The room temperature XRD measurements on all samples were consistent with mixed phases of orthorhombic and monoclinic symmetry. However, the in-plane lattice constants of these two phases differ by less than 1%, leading to minimal influence on band structure calculations. Furthermore, any impact on the experimental Fermi surface attributable to such deviations would be obscured by broadening of the bands measured by ARPES.

Therefore, the DFT calculations adopt the higher symmetry orthorhombic crystal structure with space group *Cmca* and lattice constants $a$=5.417 Å, $c$=5.468 Å, $b$=27.962 Å, with some additional relaxation of the internal coordinates. The detailed atomic coordinates are presented in Supplementary Table 1. A comprehensive plot of the DFT results for this orthorhombic structure is shown in Supplementary Fig. 4.

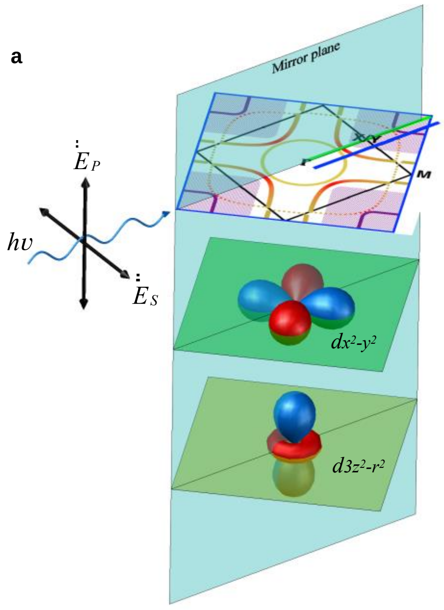


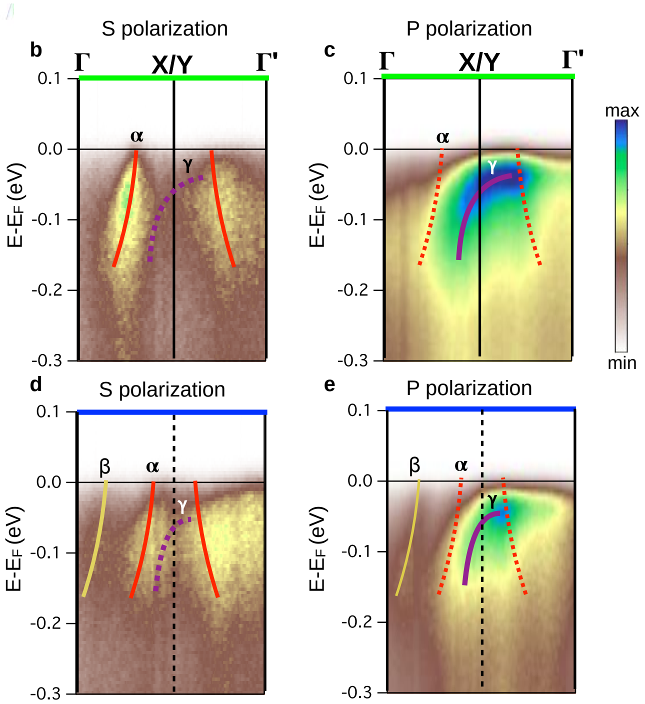


f

| | s pol. (odd) | p pol. (even) |
|---|---|---|
| $dx^2$-$y^2$ (odd) | strong | weak |
| $d3z^2$-$r^2$ (even) | weak | strong |

**Supplementary Figure 1 | Polarization dependent ARPES measurement.** (**a**) Schematic drawing of the experimental geometry and the Fermi surface map for spectra in b and c. The green line indicates the cut positions of spectra in panel **b** and **c**. The blue line indicates the cut position of spectra in panel **d** and **e**. (**b, c**) Spectra taken with s polarization light. (**d, e**) Spectra taken with p polarized light. The color scale is the same for each row of spectra. The band dispersions in each spectrum are sketched with the colored curves corresponding to the different parts in the Fermiology. The dashed curve means disfavored matrix element, and solid curve means favored matrix element. (**f**) The table describes the parity of polarization vector and orbitals with respect to the mirror plane shown in panel a and the correspondent matrix element intensity.

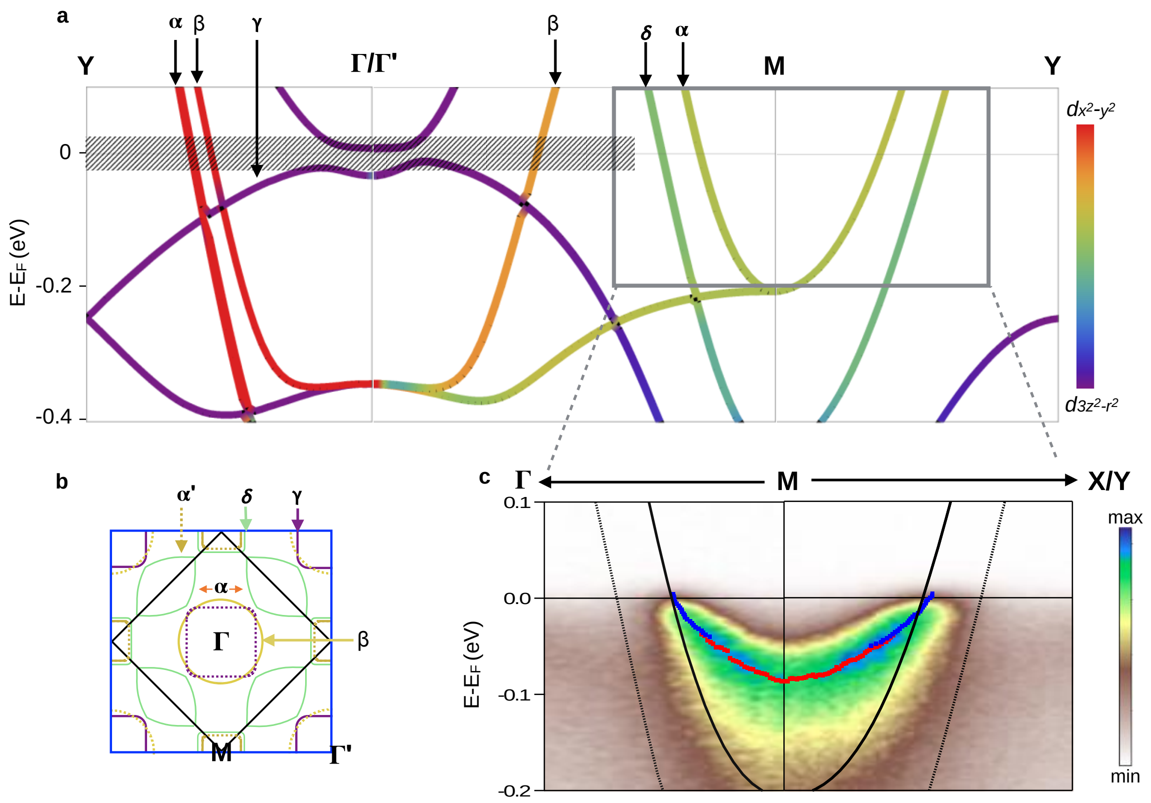


**Supplementary Figure 2 | All bands from multilayer band splitting and hybridization.** (**a**) Band structure from DFT calculation under the folded Brillouin zone. The weightings of $d_{3z^2-r^2}$ and $d_{x^2-y^2}$ orbital character are shown by the color scale plot. The hatched area indicates the blurry area from the γ band spectral weight in the Fermi surface shown in Fig. 1a1 in the paper. (**b**) The schematic drawing of the measured Fermi surface. Different parts of the Fermi surface are marked as α, β, γ, and δ. (**c**) Comparison of ARPES spectrum and the DFT band structure around the M point, where the dotted curves correspond to the outer nickel bands, the solid and dashed curves correspond to the inner nickel bands.

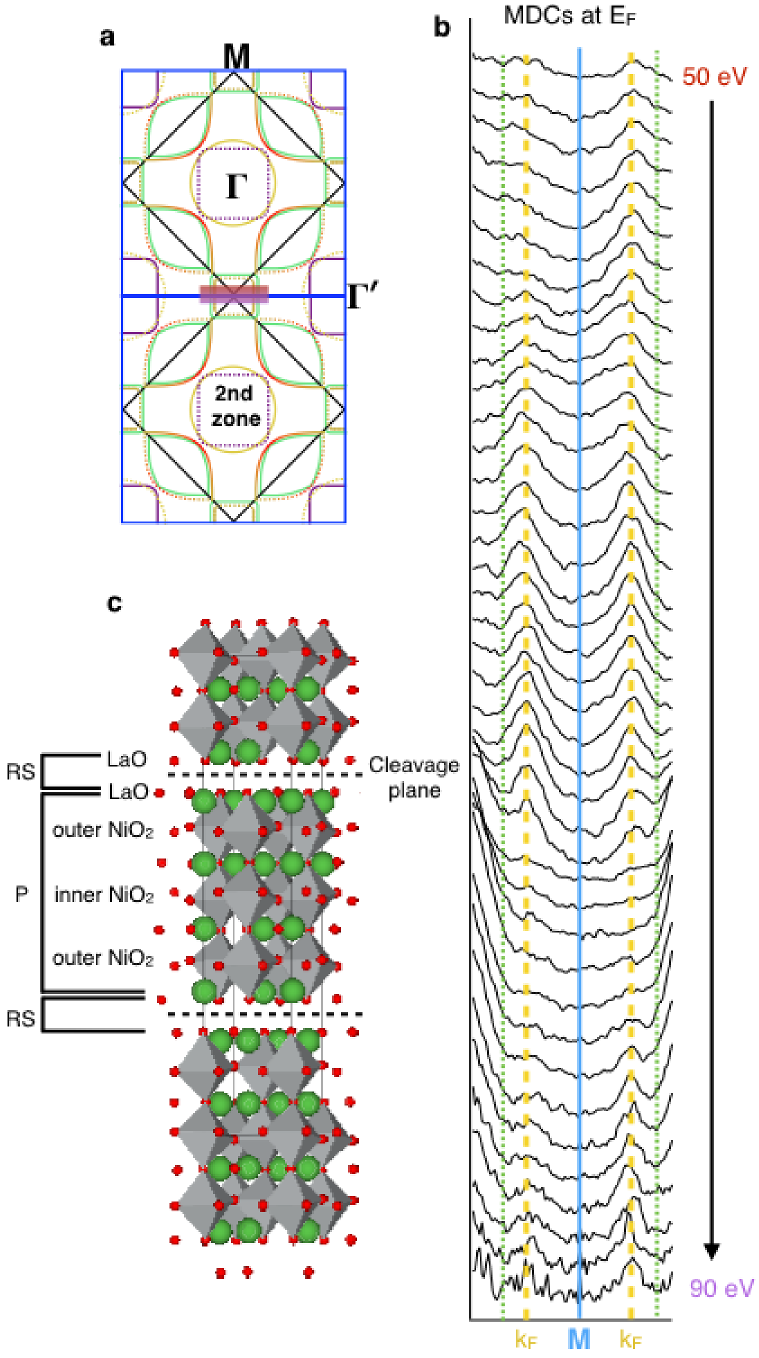


**Supplementary Figure 3 | Photon energy dependent spectra for searching for the δ band** (**a**) Schematic of the Brillouin zone including multilayer splitting. The red-purple shaded area indicates the area of the $\mathbf{k_F}$ MDC positions in panel **b.** **(b)** $\mathbf{k_F}$ MDCs of the cuts near the M point (similar to the antinodal region in cuprates.). The M point and the

$\mathbf{k_F}$ position of the band are represented by the blue solid line and the dashed yellow line respectively, whereas the green dotted line shows the expected $\mathbf{k_F}$ position of the $\delta$ band from the outer Ni-O planes, as predicted by our DFT calculation. The $\delta$ band is not clearly resolved at any photon energy. (**c**) The crystal structure of La-trilayer nickelate: P, perovskite; RS, rock salt;

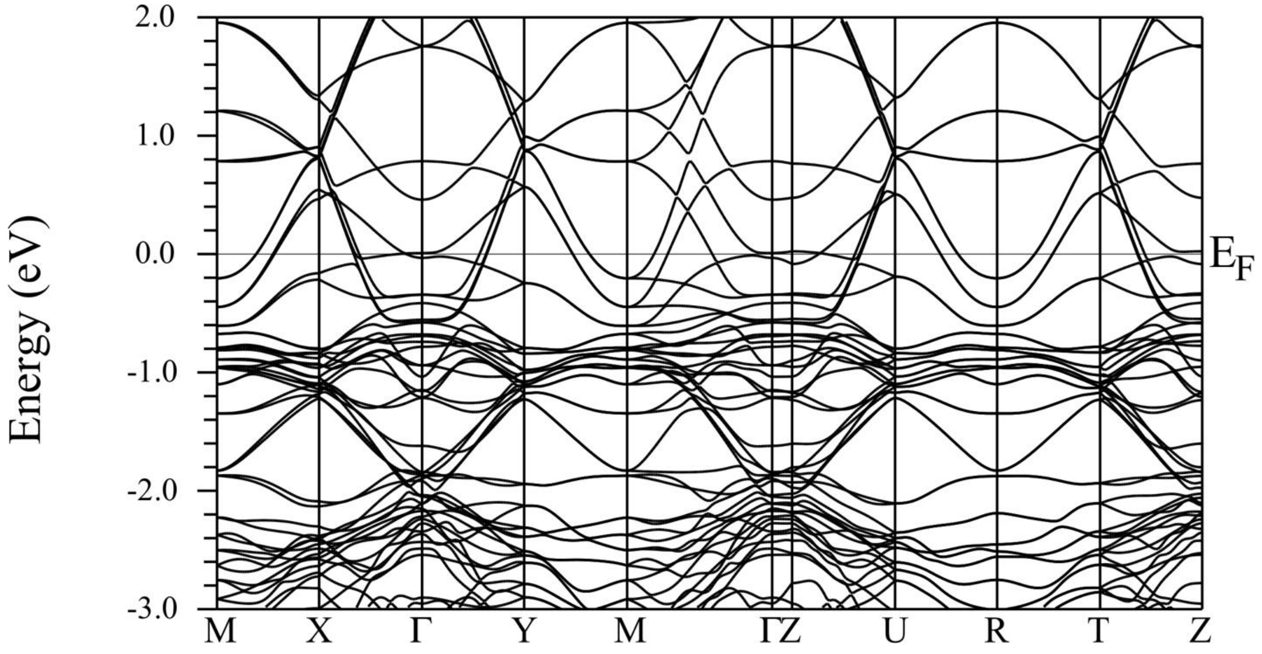


**Supplementary Figure 4 | DFT band structure of orthorhombic $La_4Ni_3O_{10}$ along more directions and over a wider energy range than is shown in the main text.**

| Atom | Wyckoff Position | Coordinates |
|---|---|---|
| **La1** | 8f | (0, 0.4326, 0.0014) |
| **La2** | 8f | (0, 0.3016, 0.0099) |
| **Ni1** | 4a | (0, 0, 0) |
| **Ni2** | 8f | (0, 0.1394, 0.0024) |
| **O1** | 8e | (0.25, 0.4921, 0.25) |
| **O2** | 8f | (0, 0.0704, 0.0501) |
| **O3** | 8e | (0.25, 0.3664, 0.25) |
| **O4** | 8f | (0, 0.2161, 0.9641) |
| **O5** | 8e | (0.25, 0.1461, 0.25) |

**Supplementary Table 1 | The detailed atomic coordinates from the DFT calculation.**

Supplementary References


1. Cao, Y. *et al.* Mapping the orbital wavefunction of the surface states in three-dimensional topological insulators. *Nat. Phys.* **9**, 499–504 (2013).
2. Emori, M. *et al.* Polarization-dependent ARPES measurement for valence band of anatase $TiO_2$. *Solid State Commun.* **188**, 15–18 (2014).
3. Müller, R. *et al.* Fermi surface map of the single-layer Bi-cuprate $Bi_2Sr_{2-x}La_xCuO_{6+\delta}$ at optimal doping. *Journal of Superconductivity* **14**, 659-668 (2001).
4. Zhang, Y. *et al.* Symmetry breaking via orbital-dependent reconstruction of electronic structure in detwinned NaFeAs. *Phys. Rev. B* **85**, 085121 (2012)
